# Aberrant Cerebral Blood Flow in Response to Hunger and Satiety in Women Remitted from Anorexia Nervosa

**DOI:** 10.3389/fnut.2017.00032

**Published:** 2017-07-19

**Authors:** Christina E. Wierenga, Amanda Bischoff-Grethe, Grace Rasmusson, Ursula F. Bailer, Laura A. Berner, Thomas T. Liu, Walter H. Kaye

**Affiliations:** ^1^Department of Psychiatry, University of California San Diego, La Jolla, CA, United States; ^2^Division of Biological Psychiatry, Department of Psychiatry and Psychotherapy, Medical University of Vienna, Vienna, Austria; ^3^Department of Radiology, University of California San Diego, La Jolla, CA, United States

**Keywords:** anorexia nervosa, hunger, cerebral blood flow, homeostatic regulation, energy metabolism, arterial spin labeling

## Abstract

The etiology of pathological eating in anorexia nervosa (AN) remains poorly understood. Cerebral blood flow (CBF) is an indirect marker of neuronal function. In healthy adults, fasting increases CBF, reflecting increased delivery of oxygen and glucose to support brain metabolism. This study investigated whether women remitted from restricting-type AN (RAN) have altered CBF in response to hunger that may indicate homeostatic dysregulation contributing to their ability to restrict food. We compared resting CBF measured with pulsed arterial spin labeling in 21 RAN and 16 healthy comparison women (CW) when hungry (after a 16-h fast) and after a meal. Only remitted subjects were examined to avoid the confounding effects of malnutrition on brain function. Compared to CW, RAN demonstrated a reduced difference in the Hungry − Fed CBF contrast in the right ventral striatum, right subgenual anterior cingulate cortex (*p*_corr_ < 0.05) and left posterior insula (*p*_unc_ < 0.05); RAN had decreased CBF when hungry versus fed, whereas CW had increased CBF when hungry versus fed. Moreover, decreased CBF when hungry in the left insula was associated with greater hunger ratings on the fasted day for RAN. This represents the first study to show that women remitted from AN have aberrant resting neurovascular function in homeostatic neural circuitry in response to hunger. Regions involved in homeostatic regulation showed group differences in the Hungry − Fed contrast, suggesting altered cellular energy metabolism in this circuitry that may reduce motivation to eat.

## Introduction

The motivation to eat in humans is a complex process influenced by intrinsic mechanisms relating to hunger and satiety, and extrinsic mechanisms based on the appetitive incentive value of food (1). Eating disorders defy homeostatic drives, suggesting that pathological eating may result from a disruption in these mechanisms. Anorexia nervosa (AN) is characterized by severe food restriction leading to significantly low body weight along with an intense fear of gaining weight and a distorted body image (2). The etiology of pathological eating in AN remains poorly understood, though recent research has implicated neural substrates underlying altered reward processing (3–5), cognitive control (6–8), and interoception (9, 10).

Appetite regulation involves the integration of multiple physiological signals regulating energy balance with cognitive processes supporting motivation and reward. Homeostatic and hedonic brain regions, including the hypothalamus, amygdala, striatum, orbitofrontal cortex, insula, and anterior cingulate cortex (ACC), are tightly interconnected and form a network that controls feeding behavior (1, 11, 12). The hypothalamus, a homeostatic center, regulates metabolic processes, including hunger and food intake, with motivation-reward systems associated with the hedonic drive to eat (1, 11). The insula, a key area in the neural control of intrinsic homeostatic processes, serves as the primary gustatory and interoceptive region in the human cortex (13). The striatum is involved in anticipation and detection of reward (14). It is activated in response to hunger (1) and less so in response to satiety (15–17), suggesting that one of its roles, along with areas such as the insula, is to motivate eating (17). The ventromedial prefrontal cortex (vmPFC), which includes the subgenual cingulate (sgACC; Brodmann area 25) and rostral ACC, acts as a visceromotor area (18–20) and governs the hypothalamus, amygdala, and insula. Together in a coordinated effort, these brain regions interpret the metabolic state of an individual and regulate appetite, motivation, and reward processing (11).

Cerebral blood flow (CBF) is tightly coupled with brain metabolism underlying cognition by increasing local delivery of oxygen and glucose to support neural function and remove metabolic by-products (21). This hemodynamic neurovascular coupling ensures a strong increase of CBF and neuronal glucose uptake with enhanced neural activity (22). Thus, CBF is an indirect marker of neuronal function and is most commonly measured with positron emission tomography (PET) ^15^O-labeled water, single-photon emission tomography (SPECT), and more recently, arterial spin labeling (ASL) functional magnetic resonance imaging (fMRI). Hunger and food intake are associated with localized changes in CBF. In healthy adults, PET ^15^O-labeled water studies reveal hunger increases CBF in homeostatic and gustatory brain regions including the hypothalamus, insula, striatum, ACC, amygdala, limbic/paralimbic regions (orbitofrontal cortex, parahippocampal gyrus, and hippocampus), thalamus, precuneus, and cerebellum (1, 12). Food intake, in contrast, produces significant *decreases* in CBF in several regions including the thalamus, insula, parahippocampal gyrus, temporal cortex, and cerebellum (11, 15, 23) and increases CBF in the prefrontal cortex (1, 12, 15, 16), which is thought to reflect inhibitory effects on the hypothalamus to promote the termination of a meal. Alterations in this pattern of increased neural function when hungry and generally decreased neural function when satiated may contribute to aberrant food intake regulation in eating disorders.

Recent evidence suggests homeostatic influences on reward processing may be altered in AN, which may contribute to disordered eating. Hunger and satiety have powerful effects on reward and inhibitory processes. Hunger makes rewards more enticing, and satiety increases self-control in healthy adults (24). Imaging studies report an altered role of striatal processes in the reward modulation of hunger or eating in AN, although the mechanisms remain to be fully elucidated (3, 4). Decreased sensitivity to the motivational drive of hunger may explain the ability of individuals with AN to restrict food when emaciated and may implicate dysfunction of the homeostatic control system in AN.

The majority of studies examining cerebral perfusion in ill AN compared to controls report hypoperfusion at rest in regions of the gustatory/homeostatic circuit (25–28). Findings in recovered AN are more discrepant, with one SPECT study reporting hypoperfusion in restricting-type AN (RAN) (29), and other ASL studies of weight-restored (30) and remitted AN (31) reporting no regional differences in CBF. The heterogeneity in methods used to quantify CBF may contribute to these discrepant findings, with higher perfusion reported in ASL than SPECT (32, 33) images. Few studies have controlled for hunger/satiety when measuring brain metabolism in AN; thus, the influence of homeostatic signaling on CBF in AN remains poorly understood (26, 34).

The purpose of this study was to determine whether women with a prior history of AN have a diminished CBF response when hungry, suggesting that altered homeostatic regulation might contribute to reduced motivation to eat in AN. This study used pulsed ASL fMRI and a region of interest analysis to investigate differential CBF response at rest in healthy comparison women (CW) and RAN when hungry (after a 16-h fast) and after a meal. We examined remitted subjects to avoid the confounding effects of malnutrition on neural function. We hypothesized that RAN would show less difference in CBF between hungry and fed states than CW in homeostatic and hedonic brain regions supporting feeding behavior. A better understanding of physiological changes in AN in response to hunger and satiety could provide a brain-specific marker relevant to treatment and outcome.

## Materials and Methods

### Participants

Twenty-one RAN women (13 pure restricting subtype, 8 who also endorsed purging) were compared (Table 1) to 16 age- and weight-matched healthy CW. Remittance was defined (35) as maintaining a weight above 85% of average body weight, regular menstrual cycles, and no binge eating, purging, or restrictive eating patterns for at least 1 year prior to the study. Current and lifetime (Table S1 in Supplementary Material) comorbid *DSM-IV* Axis I disorders were assessed using either the Structured Clinical Interview for DSM-IV Axis I disorders [SCID-I (36): 8 RAN, 8 CW] or the Mini International Neuropsychiatric interview [M.I.N.I. (37): 13 RAN, 8 CW]. Eating disorder diagnosis was established using Module H of the SCID. The M.I.N.I. has been validated against the full Structured Clinical Interview for DSM diagnoses (SCID-P) and is a more time-efficient alternative to the SCID-P (37). No participants had a current DSM-IV Axis I diagnosis or took psychotropic medication within 3 months prior to the study; a history of alcohol or drug abuse or dependence 3 months prior to study; medical or neurological concerns; or conditions contraindicative to MRI. The study and the protocol were reviewed and approved by the Institutional Review Board of the University of California San Diego. All research participants provided written informed consent.

**Table 1:** Participant demographics and characteristics.

	CW (*n* = 16)	RAN (*n* = 21)	*t* or χ^2^	*p*	Cohen’s *d*

	Mean ± SEM [min–max]	Mean ± SEM [min–max]			
**Scanner**					
GE Signa Excite	7	12	χ^2^(2) = 0.65	0.52	
GE MR750	9	9			
**Characteristics**					
Age	23.9 ± 1.5 [20.0–44.0]	27.2 ± 1.7 [19.0–45.0]	*t*(35) = 1.40	0.17	0.48
Current BMI	22.4 ± 0.4 [20.0–26.0]	21.8 ± 0.3 [19.0–24.0]	*t*(35) = 1.10	0.28	0.36
Lowest BMI	20.5 ± 0.4 [16.8–23.4]	14.9 ± 0.3 [11.3–16.9]	*t*(35) = 10.87	<0.001	3.66
Estradiol (pg/mL)^a^	50.4 ± 16.0 [9.0–221.0]	40.1 ± 11.0 [5.0–187.0]	*t*(29) = 0.55	0.59	0.76
**Neuropsychiatric assessments**					
Beck Depression Inventory	0.4 ± 0.1 [0.0–1.0]	2.4 ± 0.6 [0.0–9.0]	*t*(35) = 3.23	0.004	1.22
STAI state anxiety	25.4 ± 0.9 [20.0–30.0]	29.6 ± 1.8 [20.0–46.0]	*t*(35) = 2.04	0.05	0.69
STAI trait anxiety	24.8 ± 1.0 [20.0–34.0]	29.6 ± 1.3 [21.0–42.0]	*t*(35) = 2.83	0.008	0.98
TCI harm avoidance^b^	7.6 ± 0.9 [1.0–16.0]	11.3 ± 1.3 [2.0–23.0]	*t*(34) = 2.17	0.04	0.76
TCI reward dependence^b^	17.1 ± 1.0 [9.0–24.0]	19.3 ± 0.6 [12.0–23.0]	*t*(34) = 1.95	0.06	0.65

*Entries are of the form mean ± SEM [min–max]. Statistical comparisons were either by means of Welsh *t*-tests or χ^2^ test for equality of proportions*.

*BMI, body mass index; CW, healthy comparison women; RAN, women remitted from anorexia nervosa; STAI, Spielberger State-Trait Anxiety Inventory; TCI, Temperament and Character Inventory*.

*^a^Measured on the day of the first scan to confirm menstrual status; three CW and four RAN did not complete this assessment*.

*^b^One CW did not complete this assessment*.

### Assessments

Current symptoms were assessed using the State-Trait Anxiety Inventory (37–39), the Temperament and Character Inventory (37, 39), the Beck Depression Inventory (40, 41), and the Eating Disorders Inventory (41). Participants were studied within the first 10 days (early follicular phase) of their menstrual cycle based on their self-report. At 1:30 p.m. on the day prior to the first scan, blood samples were drawn to measure baseline levels of estradiol in order to confirm participants were in the follicular phase of their menstrual cycle. Samples were not collected for three CW and three RAN. Participants also completed Likert-type scales rating anxiety and hunger ranging from 0 (not at all) to 7 (extreme) at 3:00 p.m. the day before a scan visit (baseline), and at 6:45 a.m. (awakening), 8:45 a.m. (pre-scan), and 11:00 a.m. (post-scan) the day of a scan visit.

### Experimental Design

Participants completed a resting-state whole-brain pulsed arterial spin labeling (pASL) MR scan on two visits, 24 h apart. For the hungry state, participants fasted for 16 h (i.e., starting at 4 p.m. the previous day, with *ad libitum* water permitted) prior to the scan session. During the fed state, participants consumed standardized meals on the day prior to study and a standardized breakfast [containing 30% of overall, individualized, total daily caloric needs calculated as 30 kcal/kg body weight, and averaging approximately 450–500 kcal, with a macronutrient distribution of 53% carbohydrates, 32% fat, and 15% protein (15)] 2 h prior to the 9 a.m. scan session. Participants were instructed to select food items that represented their typical breakfast, and study staff ensured the meal met the above macronutrient distribution. For the entire study, subjects were housed and provided meals by the UCSD Clinical & Translational Research Institute to ensure 100% compliance. The visit order was randomized across participants, and imaging data were collected on one of two 3-T GE scanners in the early follicular phase.

### MRI Protocol

Resting brain blood perfusion was measured with pASL using a modified flow-sensitive alternating inversion recovery sequence with both presaturation pulses and PICORE QUIPSS 2 post-inversion saturation pulses and a spiral readout with four interleaves to reduce signal dropout due to susceptibility effects (42). Imaging data were collected on one of two scanners with an 8-channel head coil: a 3-T GE Signa HDx (GE Medical Systems, Milwaukee, WI, USA), or, due to a scanner upgrade, a 3-T GE Discovery MR 750 (GE Medical Systems, Milwaukee, WI, USA). Imaging parameters of the ASL scan for both systems were: 22 cm × 22 cm field of view, a 64 × 64 matrix, 3.2 ms echo time, 2,500 ms repetition time, post-saturation and inversion times of TI1 = 600 ms and TI2 = 1,600 ms, tag thickness 10 cm, tag to proximal slice gap 1 cm, 20 5 mm axial slices, and 40 volumes for 20 tag + control image pairs (43). A scan with the 90° excitation pulse turned off for the first eight repetitions was acquired to obtain the equilibrium magnetization of cerebrospinal fluid (CSF; a 36-s scan with TR = 4 s, TE = 3.4 ms, NEX = 9). The CSF signal was used to estimate the equilibrium magnetization of blood, which in turn was used to convert the perfusion signal into calibrated CBF units (mL/100 g tissue/min). A 32-s minimum contrast scan was acquired using an eight-shot acquisition with TR = 2,000 ms, TE = 11 ms, NEX = 2 to estimate the combined transmit and receive coil inhomogeneities (44). The two images were averaged to create the minimum contrast image. The ASL image was then divided by the minimum contrast image to remove the effect of coil inhomogeneity during the CBF quantification step (45). High-resolution T1-weighted FSPGR anatomical images (Signa HDx: TR = 7.7 ms, TE = 2.98 ms, flip angle = 8°, 192 × 256 matrix, 172 1 mm sagittal slices; MR 750: TR = 8.1 s, TE = 3.17 ms, flip angle = 8°, 256 × 256 matrix, 172 1 mm sagittal slices) were obtained for subsequent spatial normalization and activation localization. Multisite imaging studies suggest that inter-participant variance far outweighs site or magnet variance (46–48). However, to control for potential differences due to magnet hardware, groups were balanced across magnets (Table 1), each participant was scanned on the same scanner model for both imaging visits, and scanner was included as a covariate in group analyses.

### MRI Preprocessing

Image processing was performed with Analysis of Functional NeuroImages (*AFNI*^1^) (49), FMRIB Software Library^2^ (*FSL*, Oxford, UK) (50), and locally created MatLab scripts. Each ASL dataset was reconstructed using the SENSE algorithm (51, 52) to reduce sensitivity to the modulations that occur between shots caused by physiological fluctuations or motion. An automated MatLab script was used to preprocess the ASL data using AFNI and FSL tools. The ASL time series was coregistered to the middle time point to minimize the effects of participant motion. For each subject, a mean ASL image was formed from the average difference of the control and tag images using surround subtraction to create an uncorrected perfusion time series, and slice timing delays were accounted for, making the inversion time (TI2) slice specific (53). This mean ASL image was then converted to absolute units of CBF (mL/100 g tissue/min) using an estimate of the equilibrium magnetization of CSF as a reference signal (54). This procedure resulted in a calibrated perfusion value for each voxel. Skull stripping of the high-resolution T1-weighted image was performed using Brain Surface Extractor (55, 56), shown to outperform other methods (57). Scans were manually edited to remove residual non-brain material when necessary. Tissue segmentation was performed using FSL’s Automated Segmentation Tool (FAST) algorithm to define CSF, gray matter (GM), and white matter (WM) regions. The high-resolution T1-weighted image and partial volume segmentations were registered to ASL space, and partial volume segmentations were downsampled to the resolution of the ASL data.

#### Corrections for Partial Volume Effects

To correct the CBF measures for partial volume effects and ensure that CBF values were not influenced by known decreased perfusion in WM or increased volume of CSF (58, 59), we used the method previously reported by Johnson and colleagues (60). These calculations assume that CSF has 0 CBF and that CBF in GM is 2.5 times greater than that in WM. The following formula was used to compute partial volume corrected CBF signal intensities: CBF_corr_ = CBF_uncorr_/(GM + 0.4 × WM). CBF_corr_ and CBF_uncorr_ are corrected and uncorrected CBF values, respectively. GM and WM are GM and WM partial volume fractions, respectively. Information from the high-resolution structural image and the FSL FAST was used to determine the tissue content of each perfusion voxel. Using AFNI, a 4.0 -mm full-width, half-maximum Gaussian filter was applied to the CBF_corr_ data. Voxels with negative intensities were replaced with 0 (61). CBF_corr_ data were registered to the MNI-152 atlas using FMRIB’s Non-linear Image Registration Tool, part of FSL and resampled to a 3 mm × 3 mm × 3 mm resolution grid. Data were then screened for data quality, and outlying values deviating by more than 3 SDs of the mean were eliminated.

### Definition of Search Regions of Interest

Restricting the search space to a small number of *a priori* ROIs is recommended for smaller clinical samples to improve power and reduce an inflated false discovery rate (62). Four bilateral ROIs associated with homeostatic regulation were selected based on prior findings (1, 11, 12) and included the hypothalamus, ventral striatum (VST), vmPFC, and insula (Figure S1 in Supplementary Material). The VST was based on known functional distinctions (63, 64) and was defined as the nucleus accumbens extending into the rostroventral caudate and ventrolateral putamen. The vmPFC was based on the Harvard-Oxford atlas and was composed of the rostral ACC, known to project to the limbic striatum (65) and subcallosal cortex (aka, sgACC). We distinguished rostral from caudal ACC by drawing a 45° line from the anterior commissure as described by Yucel et al. (66). The insula mask from the Harvard-Oxford atlas was used in its entirety. The hypothalamus was manually traced based on prior methods (67).

### MRI Statistical Analyses

To investigate whether groups differ in the magnitude of CBF change when hungry versus fed, a difference measure (Hungry − Fed) was calculated for each individual. Student’s *t*-tests using AFNI’s 3dttest++ with the −Clustsim option were used to examine group differences in the relative change in CBF between the hungry and fed conditions within the VST, ACC, insula, and hypothalamus ROIs. Each ROI, with the exception of the hypothalamus, was treated as a search region. The 3dttest++ program performs randomization of the voxel-wise *t*-tests and then feeds these randomized *t*-statistic maps into 3dClustSim directly for cluster-size threshold determination without any spatial model for the autocorrelation function (ACF) and is the approach recommended by the AFNI group (68) to address the problem of inflated false-positive rates in prior software versions (69). A peak voxel of *p* < 0.001 with a cluster threshold of *p* < 0.05 was required for significance. At the ROI level, the required minimum cluster size was 27 µL (1 contiguous voxel) for the VST, 81 µL (3 contiguous voxels) for the vmPFC, and 81 µL (3 contiguous voxels) for the insula. Due to the small size of the hypothalamus, whole-ROI Hungry − Fed CBF was calculated for each subject, and these values were submitted to a Student’s *t*-test. A secondary exploratory whole-brain voxel-wise group comparison was also conducted. At the whole-brain level, a minimum cluster volume of 702 µL (26 contiguous voxels) was required to correct for multiple comparisons at *p* < 0.05 corresponding to a voxel-level threshold of *p* < 0.001.

#### Exploratory Associations with Clinical Variables

Mean CBF was extracted from significant clusters resulting from the between group *t*-test on the within-subject Hungry − Fed contrast. Within the RAN and CW groups, exploratory Huber robust regressions (70), Bonferroni corrected to control for family-wise error, were conducted in R to examine the relationship of pre-scan hunger ratings and CBF during the Hungry and Satiated visits within each ROI. Secondary analysis examined the relationship of CBF with other clinical variables [harm avoidance, state anxiety, trait anxiety, depression, age, current body mass index (BMI), and for RAN only, lowest post-pubertal BMI].

## Results

### Demographics and Baseline Clinical Assessments

Participants did not differ in terms of age or BMI (Table 1). Using standardized assessment instruments (5, 35), RAN had elevated levels of depression (though not clinically significant), trait anxiety, and harm avoidance relative to CW on admission to the study.

### Self-Report Assessments when Hungry or Satiated

**Figure 1:**
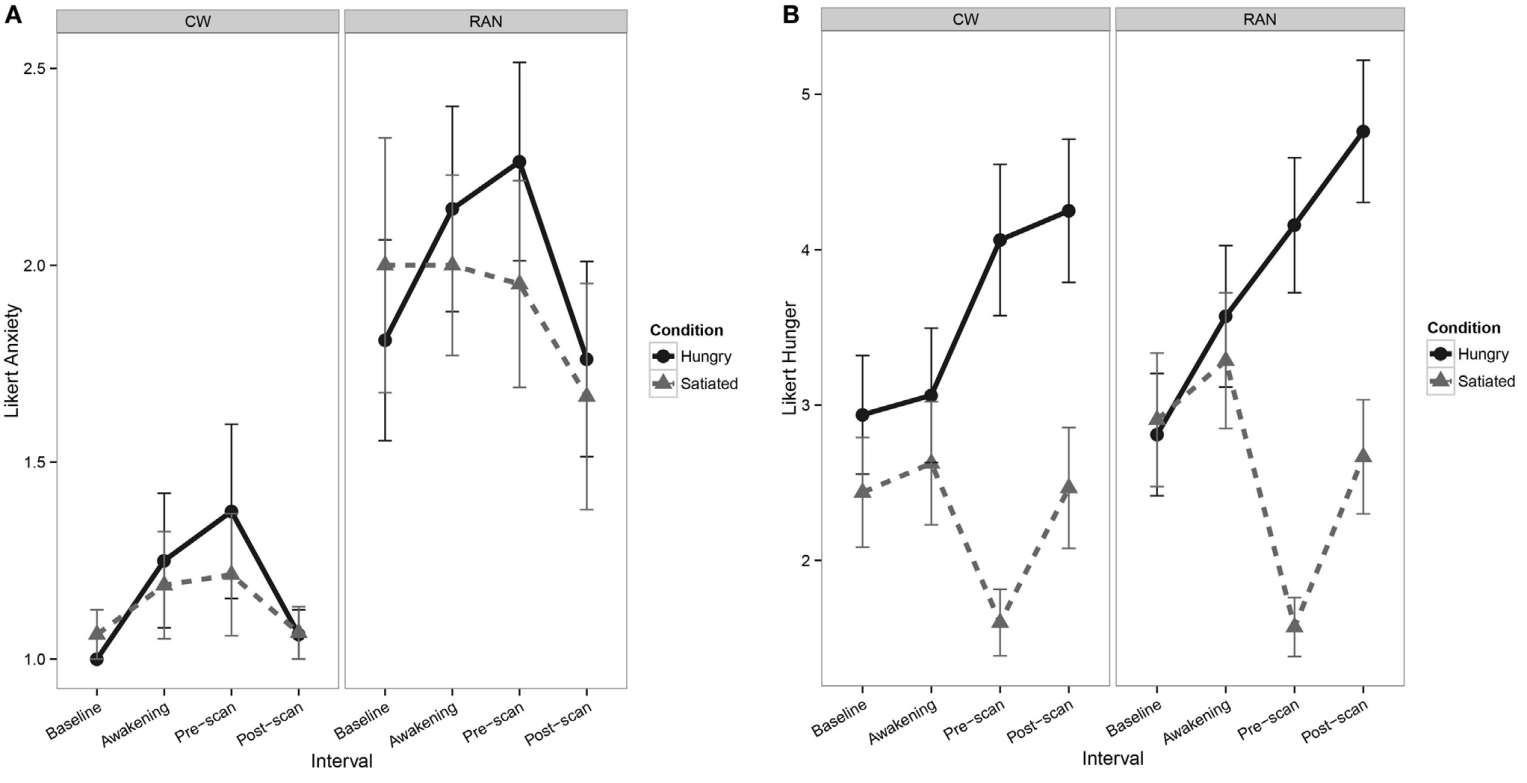
Line graphs depicting self-report Likert-type visual analog scale values. **(A)** Line graph of pre- and post-scan self-report measures of current anxiety show a main effect of Group [*F*(1,35) = 8.1, *p* = 0.007], with RAN reporting greater anxiety relative to CW [*z* = 2.56, *p* = 0.010] and interval [*F*(1,100) = 6.6, *p* = 0.011], although *post hocs* were not statistically significant. No main effect of Visit or any interactions of Group, Visit, and Interval were found. **(B)** Line graphs of pre- and post-scan self-report measures of hunger show a main effect of Visit [*F*(1,35) = 138.1, *p* = < 0.001], with participants reporting greater hunger in the Hungry condition relative to the Satiated condition [*z* = 6.04, *p* < 0.001], and a main effect of Interval [*F*(1,101) = 12.8, *p* < 0.001], although *post hocs* were not statistically significant. No main effect of Group or any interactions of Group, Visit, and Interval were found. CW, healthy comparison women; RAN, women remitted from anorexia nervosa.

At the time of scan, RAN reported greater current anxiety on a 7-point Likert-type scale when hungry or satiated relative to CW (Figure 1A). All participants reported greater hunger during the Hungry condition relative to the Satiated condition (Figure 1B).

### ROI Analysis

**Figure 2:**
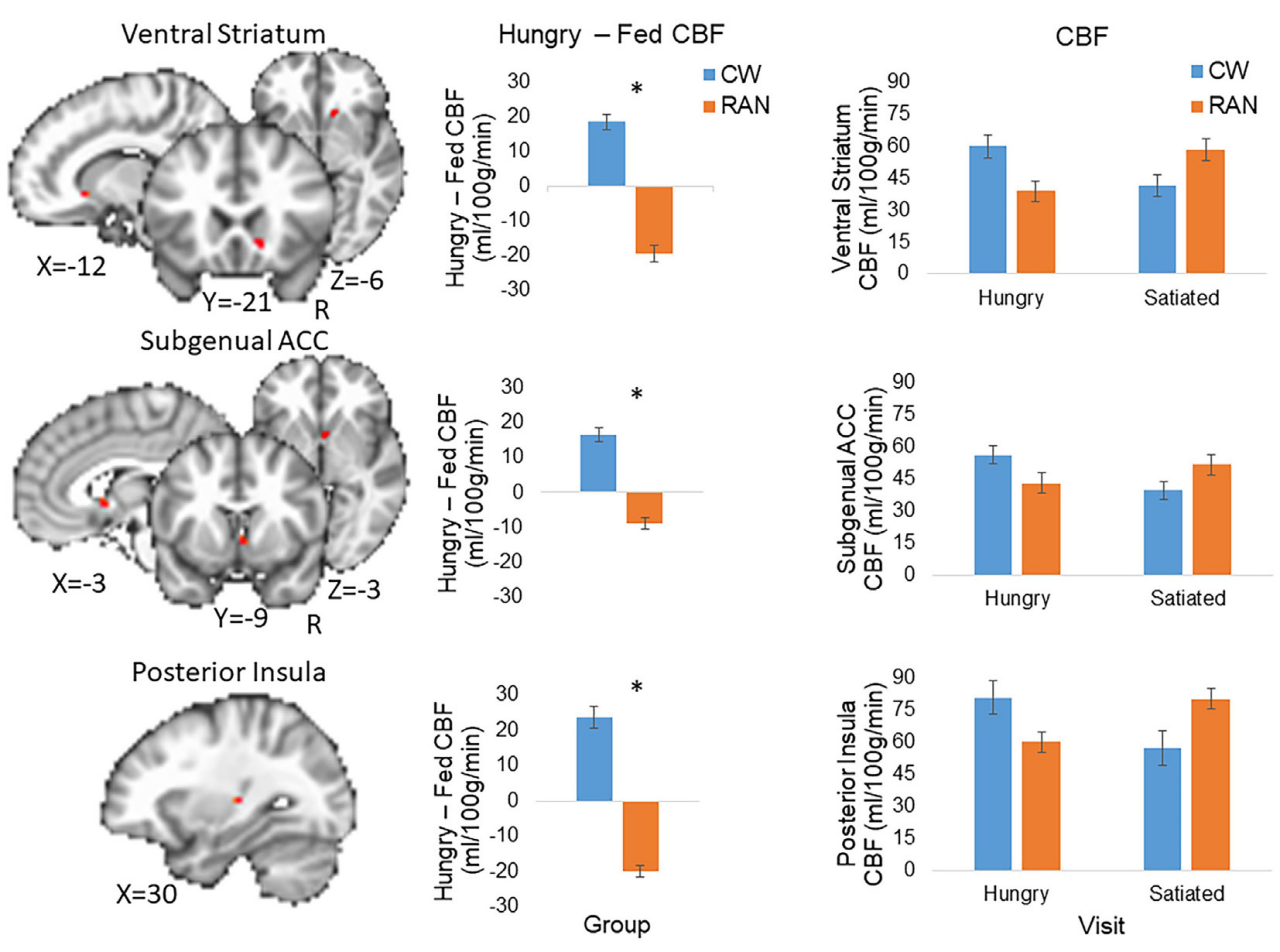
Altered CBF response to hunger in RAN. Student’s *t*-test results revealed between group differences in the Hungry − Fed contrast within regions of interest (voxel-wise threshold *p* < 0.001, cluster size corrected for multiple comparisons at *p* < 0.05). Error bars represent the SE for each group; *p < 0.05. Statistical maps are overlaid onto the MNI152 Harvard-Oxford Atlas 3 mm standard brain. CW, healthy comparison women; RAN, women remitted from anorexia nervosa; VST, ventral striatum; ACC, anterior cingulate cortex; CBF, cerebral blood flow; R, right.

Because statistical tests of group differences in Hungry − Fed CBF evaluated *a priori* hypotheses, we report all results and indicate whether or not the significant clusters survived adjustment to *p*-values for the small number of statistical tests performed (71, 72). Effect sizes are provided in addition to *p*-values (magnitude of *d*: small 0.2, medium 0.5, and large 0.8) (73). Within our ROI search regions, significant group differences in the Hungry − Fed CBF contrast measure were found in the right VST and right subgenual ACC (*p*_corr_ < 0.05), and in the left posterior insula (*p*_unc_ < 0.05) (Figure 2; Table 2). CW had greater CBF when hungry versus fed, whereas RAN had reduced response when hungry versus fed. No group differences were detected in the hypothalamus.

**Table 2:** *T*-test results within regions of interest demonstrating a group difference in the Hungry − Fed contrast for cerebral blood flow (CBF).

ROI	Subregion	R/L	Volume (μL)	RL	AP	IS	Peak *t*	*p*	Cohen’s *d*^a^
Ventral striatum		R	108	−12	−21	−6	3.86	<0.001	0.55
Ventromedial prefrontal cortex	Subgenual anterior cingulate cortex (ACC), adjacent to the caudate head	R	108	−3	−9	−3	3.70	<0.001	0.22
Insula^b^	Posterior insula	L	54	30	24	6	3.98	<0.001	0.14

*Small volume correction was determined with Monte–Carlo simulations (via AFNI’s 3dClustSim) to guard against false positives. Coordinates are reported as the center of mass and are presented in RAI format*.

*^a^Cohen’s *d* values are presented for the group difference in CBF in the Hungry − Fed contrast averaged across each anatomical ROI to avoid the possibility of over-inflation by restricting analysis to significant (“non-independent”) clusters (74)*.

*^b^Insula cluster did not meet cluster-size threshold of 81 µL to control for multiple comparisons*.

*RL, right-left direction; AP, anterior–posterior direction; IS, inferior–superior direction; L, left; R, right; CW, healthy comparison women; RAN, women remitted from anorexia nervosa*.

### Voxel-Wise Analysis

Exploratory whole-brain voxel-wise analysis (voxel-wise threshold *p* < 0.001, cluster size >702 μL corrected for multiple comparisons at *p* < 0.05) did not detect any significant group differences in the Hungry − Fed CBF contrast.

### Relation to Other Clinical Variables

**Figure 3:**
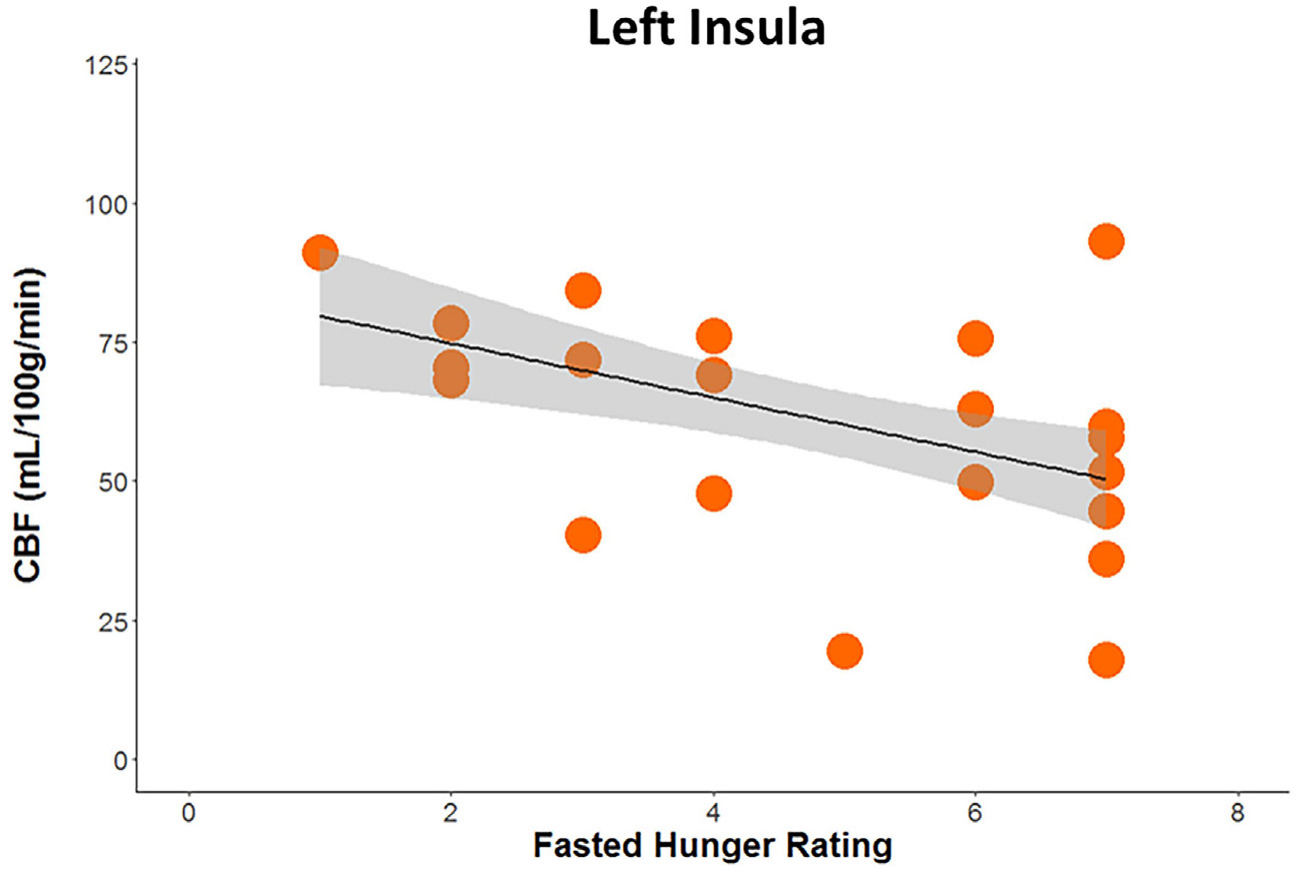
Plot demonstrating results of robust regression depicting the association between pre-scan hunger ratings and cerebral blood flow when hungry within the left posterior insula in women remitted from anorexia nervosa (*p* < 0.05, Bonferroni corrected for multiple comparisons).

Huber robust regression analysis revealed that greater pre-scan hunger ratings were associated with decreased CBF in the left insula (*t* = −2.67, *p* = 0.008) only in RAN when hungry, Bonferonni corrected for 2 conditions × 3 regions of interest (Figure 3; Table S2 in Supplementary Material). The interaction of the regression slopes for hunger ratings and insula CBF when hungry with group trended toward significance [*F*(2,36) = 3.2, *p* = 0.054], raising the possibility that this difference between RAN and healthy controls was driven by the hungry RAN women, which might reflect a disconnect between the hunger sensation and brain response in AN. None of the secondary analyses between CBF and other clinical variables reached significance after controlling for multiple comparisons (Table S2 in Supplementary Material).

## Discussion

This represents the first study to show that women remitted from AN have aberrant CBF at rest in homeostatic and hedonic food motivation pathways in response to hunger. Using pulsed ASL, we reproduced previous PET findings (12) in a larger sample of healthy women and found that hunger was associated with increased CBF in regions involved in appetite regulation, including the right VST, subgenual ACC, and left posterior insula. In contrast, RAN showed reduced CBF when hungry relative to when satiated in these same regions. These regions have been implicated consistently in studies of AN (3–10). Interestingly, there was a suggestion that for the RAN group, diminished CBF in the left posterior insula when hungry was associated with increased ratings of hunger. Why hunger in RAN may be associated with reduced CBF in regions responsible for valuating rewarding and motivated behavior is not well understood, but this aberrant response may shed light on why RAN lack the ability to appropriately evaluate food reward or to eat when hungry.

Findings in RAN of a decreased Hungry − Fed CBF response in the posterior insula (uncorrected), VST, and subgenual ACC, and associations between diminished CBF in the posterior insula when hungry and increased hunger ratings further support the potential role of the insula in integrating homeostatic information (e.g., hunger) and reward value with feeding behavior (75). The dorsal posterior insula is the primary interoceptive representation of the body’s homeostatic sensations, including hunger (13). This information is re-represented in the anterior insula, where interoceptive information is integrated with motivational and emotional processes, supporting feeling states, and giving rise to conscious visceral perception of homeostatic states (76, 77). Clinically, patients with AN report poor interoceptive awareness (78) and demonstrate interoceptive prediction errors during meal anticipation (10). Disturbed interoceptive awareness of hunger could play a role in assessing body states and responding to hunger cues. Findings of altered CBF in the posterior insula support the notion that AN might suffer from a fundamentally and physiologically altered sense of physical state, and that brain circuits may misperceive signals regarding hunger (78). This is consistent with several fMRI studies reporting altered insula function in RAN (9, 79, 80).

Between group CBF differences in the striatum, with reduced Hungry − Fed CBF for RAN, support the notion that alterations in metabolic function when hungry may contribute to food avoidance and/or impaired reward learning in AN (20). Many individuals with AN respond in a fearful or avoidant manner to salient rewards, such as highly palatable food, rather than experiencing pleasure, and computational modeling studies show increased learning from punishment as opposed to reward in RAN (81). We have previously proposed that individuals with AN may have an intrinsic sensitivity to coding salient stimuli, such as food, as aversive or risky, rather than rewarding, that overrides the influences of hunger (82). Abnormalities of structure and function within the extended “visceromotor” network (involving vmPFC and anatomically related limbic, striatal, thalamic, and basal forebrain structures) impair this network’s roles in cognitive processes such as reward learning and may dysregulate visceral, behavioral, and cognitive responses to emotional stimuli and stress (20). Prior fMRI studies of reward processing in RAN show underactive reward valuation circuitry (e.g., VST) to immediate salient stimuli when hungry (3, 5, 82). Our results of CBF differences at rest suggest that food restriction in AN may be due to difficulty in appropriately binding, scaling, or discriminating emotionality and reward in response to salient stimuli due to homeostatic dysfunction. Our data, and those of others (83) suggest that AN can sense their hunger state when actively ill and recovered, and they can accurately assess palatability of foods and sweet taste (79, 84). Consistent with prior findings of an aberrant relationship between ghrelin signaling and neural reward response in AN, our data raise the intriguing hypothesis that AN may have difficulty translating homeostatic signals associated with energy balance into motivated eating behavior, and this may not normalize in recovery. In other words, while RAN appear to experience hunger accurately, it is possible that hunger is less effective at stimulating brain mechanisms supporting the adaptive drive to eat. This suggests that individuals who recover may rely on other strategies to motivate eating (e.g., cognitive control or external guides).

What is the meaning of altered CBF in this study? Because vascular supply is varied locally in the brain in correspondence with local variations of functional activity, CBF is generally thought to be a surrogate marker of energy delivery and use that is coupled with neuronal activity and metabolism. ASL measures CBF in the capillary bed, and thus changes in CBF may also reflect the function of astrocytes, given their critical role in mediating the coupling of neuronal activity with metabolic and vascular responses (85). Astrocytic end feet wrap around the endothelium of capillaries and *via* this contact, they can influence CBF and control the transport of nutrients, such as glucose, in and out of the brain to ensure proper brain homeostasis and support neurons metabolically (85, 86). Recent evidence suggests astrocytes, similar to neurons, respond directly to multiple nutrient and endocrine signals and, in turn, contribute to adjusting central nervous system control of systemic metabolism according to nutrient availability (87). This raises the question of whether our findings reflect an aberrant neural-astrocyte response to peptides, enzymes, or metabolic hormones that disrupt energy balance or hunger signaling in AN. Although we were unable to collect plasma glucose, insulin, or hormones (e.g., leptin, ghrelin) on a sufficient number of participants, there is evidence in women with AN of altered fMRI activation of neural circuits involved in food motivation that is associated with abnormal levels of appetite-regulating hormones (88).

Decreased CBF at rest in RAN when hungry compared to when satiated may contribute to the decreased fMRI blood oxygenation level dependent (BOLD) response to food and money rewards associated with hunger in AN (3, 5). Generally interpreted as an indirect qualitative measure of neuronal activity, the fMRI BOLD signal reflects local changes in deoxyhemoglobin content, which in turn exhibits a complex dependence on changes in CBF, cerebral blood volume, and cerebral metabolic rate of oxygen consumption (22). Of these quantities, CMRO_2_ is thought to be most tightly linked to neural activity, reflecting the notion that neurons necessarily expend energy to accomplish their work (89). The positive BOLD response observed in most fMRI experiments reflects the fact that CBF increases relatively more than CMRO_2_, so that local capillary and venous blood are more oxygenated during increased brain activity. In general, the actual amplitude of the BOLD response reflects a delicate balance between the relative increases in CBF and CMRO_2_ (21). Thus, decreased BOLD response to hunger in AN may reflect impaired CBF response and/or altered CBF/CMRO_2_ coupling or a decrease in neural activity with normal CBF/CMRO_2_ coupling. However, without a *quantitative* estimate of functional changes in CMRO_2_, which can be obtained by simultaneously acquiring functional BOLD and CBF data during a cognitive task and a hypercapnic challenge (a.k.a., calibrated fMRI) (90), the impact of CBF on BOLD response in AN is not well understood.

This study has several strengths, including quantification of CBF, rigorous control of hunger and satiety, and studying well-characterized remitted subjects to avoid potential confounds of malnutrition. While ^15^O-labeled water PET is the gold standard for CBF measurement, ASL fMRI has been shown to be as reliable a technique (91, 92) with equivalent accuracy and precision and has many advantages over this invasive method that may make it preferable in clinical research. CBF measured with ASL refers to the *rate* of delivery of arterial blood to the capillary bed in brain tissue and is typically quantified in milliliters of blood per 100 g of tissue per minute (22). ASL is a non-invasive and reliable fMRI technique (58) that magnetically labels arterial water in the brain and uses it as an endogenous tracer to measure CBF. Because ASL fMRI provides a quantitative measure of CBF in the capillary bed, rather than a relative measure such as the venous BOLD fMRI signal, it has the potential to more accurately estimate the magnitude and location of neural function (21) and is less susceptible to artifactual image degradation from surrounding bony sinuses and poor spatial resolution that limits other methods (93).

Despite these strengths, limitations are important to acknowledge. ASL fMRI does not provide a direct measure of neuronal activity, and limitations in many of the existing pulse sequences (e.g., sensitivity to transit time effects, limited brain coverage, and low spatial resolution) may account for some of the discrepancies across studies. To limit multiple comparisons, we restricted our primary analyses *a priori* ROIs; it is important to recognize these regions are part of larger valuation and salience circuits involving complex cortical–striatal processes. In contrast to prior findings (15, 16), we did not find any regions of increased CBF for satiety in the control women. Methodological differences between studies could account for discrepant findings. For example, we fed participants 30% of their individualized daily caloric needs whereas others (12) have fed 50% of daily resting energy expenditure, and our participants fasted for 16 versus 36 h in other studies (15, 16). However, our methods may be more ecologically valid, as others (12) induced rather extreme states of hunger and satiation. Because difference contrasts can be difficult to interpret, we also conducted a 2 group × 2 condition linear mixed-effect model in R. Although we replicated our *t*-test results at a voxel-level *p* < 0.01 and cluster-corrected *p* < 0.05, results did not survive at the recommended per-voxel *p*-threshold of 0.001 using AFNI’s new ACF modeling approach, suggesting the study may not be sufficiently powered at the group level for this stringent correction. Follow-up studies with larger cohorts are certainly needed. Finally, without premorbid data we cannot determine whether low CBF in response to hunger is a vulnerability factor/biomarker for weight loss or whether it reflects a scar of malnutrition. This is the first study to examine the effects of hunger and satiety on CBF in RAN.

### Clinical Implications

Obesity has long been known to result from dysfunction of key biochemical, neural, and behavioral components of the homeostatic control system (94), emphasizing the physiological importance of this system in eating behavior and weight. Our data, and others, are beginning to reveal that dysfunction of the homeostatic control system may also contribute to AN. These findings offer new targets for psychological interventions. For example, developing strategies that incorporate an understanding of altered homeostatic sensitivity into behavioral management may improve treatment compliance and outcome by enhancing insight and reducing reliance on physiological signals to guide eating behavior.

## Conclusion

The capacity to adjust food intake in response to changing energy requirements is essential for survival. Eating behavior is modulated by metabolic and ingestive factors in the service of homeostasis, and current results suggest possible metabolic dysfunction associated with homeostatic regulation may explain disordered eating in AN.

## Ethics Statement

The study was conducted in compliance with the Code of Ethics of the World Medical Association (Declaration of Helsinki) and according to the IRB regulations of the University of California, San Diego, and written informed consent was obtained.

## Author Contributions

The authors made substantial contributions to the conception or design of the work (CW, A-BG, UB, LB, and WK) or the acquisition (GR), analysis (CW), or interpretation of data (CW, A-BG, UB, LB, TL, and WK) for the work; drafting the work (CW) or revising it critically for important intellectual content (A-BG, GR, UB, LB, TL, and WK); provided final approval of the version to be published (CW, A-BG, GR, UB, LB TL, and WK); and agreed to be accountable for all aspects of the work in ensuring that questions related to the accuracy or integrity of any part of the work are appropriately investigated and resolved (CW, A-BG, GR, UB, LB, TL, and WK).

## Conflict of Interest Statement

The authors declare that the research was conducted in the absence of any commercial or financial relationships that could be construed as a potential conflict of interest.

## Footnotes

^1^http://afni.nimh.nih.gov.

^2^http://fsl.fmrib.ox.ac.uk/fsl/.
