# Supplementary material for: Aberrant Cerebral Blood Flow in Response to Hunger and Satiety in Women Remitted from Anorexia Nervosa
Source: Front Nutr. 2017 Jul 19;4:32. doi: 10.3389/fnut.2017.00032 (PMC5515860; doi:10.3389/fnut.2017.00032)
Supplement: Supplementary file 1 [file Data_Sheet_1.pdf]

SUPPLEMENTAL MATERIAL

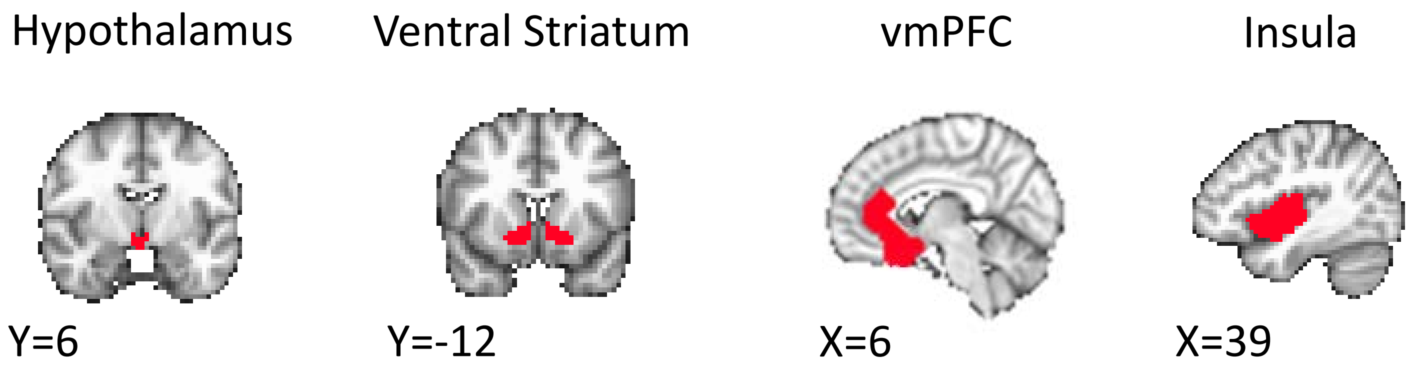

Supplementary Figure 1. Illustration of regions of interest for the examination of homeostatic regulation included the bilateral hypothalamus, ventral striatum, vmPFC (subgenual and rostral ACC), and insula derived from the Harvard-Oxford MNI Atlas.

Characteristic	CW (N=16)	RAN (N=21)	F or χ^2	p	Cohen's *d*
**Lifetime Diagnosis (n)**					
Major Depressive Disorder	0	14	$\chi^2(1,N=37)=17.16$	<0.001	
Obsessive Compulsive Disorder	0	4	$\chi^2(2,N=37)=3.41$	0.12	
Any anxiety disorder	0	7	$\chi^2(2,N=37)=6.58$	0.01	
Any alcohol	0	3	$\chi^2(2,N=37)=2.49$	0.24	
WCST Perseverative Errors[a]	7.5 ± 3.8 [4.0-19.0]	14.9 ± 2.3 [4.0-45.0]	t(33)=2.06	0.01	0.59
WCST Categories Completed[a]	6.0 ± 0.0 [6.0-6.0]	5.0 ± 0.4 [0.0-6.0]	t(33)=2.04	0.05	1.05
WASI IQ[b]	114.8 ± 2.8 [96.0-136.0]	111.9 ± 3.0 [85.0-133.0]	t(34)=0.67	0.51	0.23
YBOCS Current Total	0.0 ± 0.0 [0.0-0.0]	0.3 ± 0.2 [0.0-3.0]	t(35)=1.55	0.13	0.83
YBOCS Whole Lifetime Total	0.0 ± 0.0 [0.0-0.0]	8.3 ± 2.0 [0.0-32.0]	t(35)=3.10	0.004	1.54

Supplementary Table 1. Participant neurocognitive and psychiatric characteristics. Entries are of the form mean +/- SEM [min-max]. Statistical comparisons were either by means of Welsh t-tests or χ^2 test for equality of proportions. CW: healthy comparison women; RAN: women remitted from anorexia nervosa; RBN: women remitted from bulimia nervosa; WCST: Wisconsin Card Sorting Task; WASI: Wechsler Adult Intelligence Scale; YBOCS: Yale-Brown Obsessive-Compulsive Scale. Any anxiety disorder defined as having had at least one prior episode of panic disorder, phobia, post-traumatic stress disorder, generalized anxiety disorder, or any anxiety disorder NOS. Any alcohol defined as any history of abuse or dependent per DSM-IV criteria. Note: [a]one CW and 1 RAN did not complete this assessment; [b]one CW did not complete this assessment.

Predictor	Hunger Ratings		TCI Harm Avoidance		STAI State Anxiety		STAI Trait Anxiety		BDI		Age		Current BMI		Lowest BMI	
	t	*p*	t	*p*	t	*p*	t	*p*	t	*p*	t	*p*	t	*p*	t	*p*
RAN																
VST CBF Hungry	-.30	.77	.30	.76	-1.2	.24	-.45	.65	.30	.76	.02	.99	.10	.92	-1.0	.29
VMPFC CBF Hungry	-.78	.44	-.24	.81	-1.3	.20	-.15	.88	-.35	.72	.18	.85	-.64	.52	-2.87	.004
Insula CBF Hungry	-2.7	.008	1.3	.20	.58	.56	1.9	.05	-.34	.73	.21	.83	.44	.66	.33	.74
VST CBF Fed	-1.3	.20	-.53	.60	-1.3	.19	-1.6	.11	.04	.97	.30	.76	1.3	.19	-1.3	.21
VMPFC CBF Fed	-.85	.40	.32	.75	-.46	.64	-.92	.36	-.04	.97	.74	.46	2.1	.04	-1.8	.07
Insula CBF Fed	-.04	.97	.35	.73	-1.8	.07	-.36	.72	-1.1	.26	.32	.74	.71	.48	-.23	.82
CW																
VST CBF Hungry	.74	.46	.59	.55	-.32	.75	.08	.93	.73	.47	-.22	.78	1.5	.13	--	--
VMPFC CBF Hungry	1.9	.06	1.1	.28	1.7	.09	.70	.48	.48	.63	-.25	.80	.83	.40	--	--
Insula CBF Hungry	-.28	.78	.17	.86	-.13	.90	.98	.33	-.06	.94	-.07	.94	2.7	.006	--	--
VST CBF Fed	-.01	.99	1.0	.31	1.0	.30	1.3	.20	1.1	.29	.18	.86	1.1	.26	--	--
VMPFC CBF Fed	-.22	.83	.84	.40	1.1	.29	1.4	.17	.29	.77	-2.2	.03	.69	.49	--	--
Insula CBF Fed	.20	.84	.37	.72	.21	.84	2.4	.02	.24	.81	-.13	.20	1.8	.08	--	--

Supplementary Table 2. Associations between CBF and hunger ratings and clinical variables resulting from robust regression. TCI = Temperament and Character Inventory; STAI = Spielberger State Trait Anxiety Inventory; BDI = Beck Depression Inventory; BMI = body mass index; RAN = remitted anorexia nervosa; CW = control women; VST = ventral striatum; VMPFC = ventromedial prefrontal cortex; CBF = cerebral

blood flow. Significance after Bonferroni correction for multiple comparisons determined at $p<.0.008$ for hunger ratings (a priori) and $p<.001$ for clinical variables (exploratory).
