# Supplementary material for: Aberrant Cerebral Blood Flow in Response to Hunger and Satiety in Women Remitted from Anorexia Nervosa
Source: Front Nutr. 2017 Jul 19;4:32. doi: 10.3389/fnut.2017.00032 (PMC5515860; doi:10.3389/fnut.2017.00032)
Supplement: Supplementary file 2 [file table_1.pdf]

Characteristic	CW (N=16)	RAN (N=21)	F or χ^2	p	Cohen's *d*
**Lifetime Diagnosis (n)**					
Major Depressive Disorder	0	14	χ^2(1,N=37)=17.16	<0.001	
Obsessive Compulsive Disorder	0	4	χ^2(2,N=37)=3.41	0.12	
Any anxiety disorder	0	7	χ^2(2,N=37)=6.58	0.01	
Any alcohol	0	3	χ^2(2,N=37)=2.49	0.24	
WCST Perseverative Errors[a]	7.5 ± 3.8 [4.0-19.0]	14.9 ± 2.3 [4.0-45.0]	t(33)=2.06	0.01	0.59
WCST Categories Completed[a]	6.0 ± 0.0 [6.0-6.0]	5.0 ± 0.4 [0.0-6.0]	t(33)=2.04	0.05	1.05
WASI IQ[b]	114.8 ± 2.8 [96.0-136.0]	111.9 ± 3.0 [85.0-133.0]	t(34)=0.67	0.51	0.23
YBOCS Current Total	0.0 ± 0.0 [0.0-0.0]	0.3 ± 0.2 [0.0-3.0]	t(35)=1.55	0.13	0.83
YBOCS Whole Lifetime Total	0.0 ± 0.0 [0.0-0.0]	8.3 ± 2.0 [0.0-32.0]	t(35)=3.10	0.004	1.54

Supplementary Table 1. Participant neurocognitive and psychiatric characteristics. Entries are of the form mean +/- SEM [min-max]. Statistical comparisons were either by means of Welsh t-tests or χ^2 test for equality of proportions. CW: healthy comparison women; RAN: women remitted from anorexia nervosa; RBN: women remitted from bulimia nervosa; WCST: Wisconsin Card Sorting Task; WASI: Wechsler Adult Intelligence Scale; YBOCS: Yale-Brown Obsessive-Compulsive Scale. Any anxiety disorder defined as having had at least one prior episode of panic disorder, phobia, post-traumatic stress disorder, generalized anxiety disorder, or any anxiety disorder NOS. Any alcohol defined as any history of abuse or dependent per DSM-IV criteria. Note: [a]one CW and 1 RAN did not complete this assessment; [b]one CW did not complete this assessment.
