# Supplementary material for: Aberrant Cerebral Blood Flow in Response to Hunger and Satiety in Women Remitted from Anorexia Nervosa
Source: Front Nutr. 2017 Jul 19;4:32. doi: 10.3389/fnut.2017.00032 (PMC5515860; doi:10.3389/fnut.2017.00032)
Supplement: Supplementary file 3 [file table_2.pdf]

ROI	Subregion	R/L	Volume (μL)	RL	AP	IS	Peak t	p	Cohen's d±
Ventral Striatum		R	108	-12	-21	-6	3.86	<0.001	0.55
vmPFC	Subgenual ACC, adjacent to the caudate head	R	108	-3	-9	-3	3.70	<0.001	0.22
*Insula	Posterior Insula	L	54	30	24	6	3.98	<0.001	0.14

Table 2. T-test results within regions of interest demonstrating a group difference in the Hungry – Fed contrast for CBF. Small volume correction was determined with Monte-Carlo simulations (via AFNI's 3dClustSim) to guard against false positives. Coordinates are reported as the center of mass and are presented in RAI format. ±Cohen's d values are presented for the group difference in CBF in the Hungry – Fed contrast averaged across each anatomical ROI to avoid the possibility of over-inflation by restricting analysis to significant ("non-independent") clusters (see 94). *Insula cluster did not meet cluster-size threshold of 81 μl to control for multiple comparisons, despite a large effect size. RL: right-left direction; AP: anterior-posterior direction; IS: inferior-superior direction; L: left; R: right; CW: healthy comparison women; RAN: women remitted from anorexia nervosa.
